# Concentration with Nanofiltration of Red Wine Cabernet Sauvignon Produced from Conventionally and Ecologically Grown Grapes: Effect on Phenolic Compounds and Antioxidant Activity

**DOI:** 10.3390/membranes11050322

**Published:** 2021-04-28

**Authors:** Ivana Ivić, Mirela Kopjar, Dubravko Pichler, Ivana Buljeta, Anita Pichler

**Affiliations:** 1 Faculty of Food Technology Osijek, Josip Juraj Strossmayer University, F. Kuhača 18, 31000 Osijek, Croatia; iivic@ptfos.hr (I.I.); mirela.kopjar@ptfos.hr (M.K.); ibuljeta@ptfos.hr (I.B.); 2 Water Supply—Osijek, Poljski Put 1, 31000 Osijek, Croatia; dubravko.pichler@vodovod.com

**Keywords:** conventional Cabernet Sauvignon, ecological Cabernet Sauvignon, nanofiltration process, phenolic compounds, antioxidant activity, colour

## Abstract

The aim of this study was to investigate the influence of different operating conditions (four pressures: 2.5, 3.5, 4.5 and 5.5 MPa; two temperature regimes: with and without cooling) and wine type on phenolic compounds retention during the nanofiltration process of two Cabernet Sauvignon red wines (conventionally and ecologically produced). The nanofiltration process was conducted on Alfa Laval LabUnit M20 with plate module and six NF M20 membranes. In initial wines and obtained retentates, total polyphenol and flavonoid contents, monomeric anthocyanins content, antioxidant activity, individual phenolic compounds and CIELab colour parameters were determined. A loss of total phenolic compounds and decrease in antioxidant activity was observed in all retentates comparing to initial wine. However, retentate cooling and higher pressure increased their retention. Besides processing parameters, individual phenolic compound retention depended on several factors, such as the wine type, chemical properties of compounds and membrane type, and their combinations. Different chemical composition of initial conventional and ecological wine influenced the retention of individual compounds.

## 1. Introduction

Recent viticulture methods show a growing trend towards the production of wine with minimum or no chemical residues. This type of grape production is called ecological or organic viticulture and differs from the conventional process in replacing chemical fertilizers, pesticides, insecticides and other artificial additives with organic ones, such as natural manure [1]. Avoiding chemical substances in vineyards enables the production of wine with no chemical residues, minimizing their negative impact on human health and the environment. The main goal is to maintain the biological activity of soil (chemical substances can disrupt the content of essential minerals) [2,3]. In order to ensure minimum soil disturbance and the collection of the most ripe and healthy grapes, ecological viticulture avoids the use of machinery and grapes are collected by hand [2]. Furthermore, the conversion of conventional to ecological viticulture requires special soil pre-treatment through six to eight years, such as natural composting, clean water, rotations, etc., before it becomes suitable for ecological viticulture [4]. This is followed by the accreditation procedure and the vineyard acquires a certificate with precise localisation and starting date [3,5].

Viticulture methods, along with environmental conditions and vinification techniques, greatly affect the chemical composition and phenolic profile of grapes and wine that is produced from it. Several studies [2,6,7,8] have reported that the phenolic profile of ecologically produced wine differs from the phenolic profile of conventionally produced ones, comparing the same grape variety. Phenolic compounds originate from the seeds and skins of grapes, and they are transferred into must during crushing, maceration or fermentation [9]. Therefore, the grape maturity and health, climate, the use of fertilizers or other adjuvants, harvest conditions and vinification techniques greatly affect the final concentrations of the mentioned compounds [10].

Phenolic compounds in wine include a large group of various compounds that are divided into non-flavonoids (stilbenes, hydroxybenzoic and hydroxycinnamic acids) and flavonoids (flavonols, anthocyanins and tannins). The concentration of total phenolic compounds in red wine can vary between 1800 and 3000 mg/L depending on wine variety and production methods [11]. They contribute to wine colour, bitterness and astringency, but they also act as antioxidants protecting wine and its consumers from oxidative stress and preventing various diseases [11,12] due to their antimicrobial, antioxidant and/or anti-inflammatory effects [13]. Besides pre-fermentation and fermentation procedures during winemaking, post-fermentation also has a great influence on phenolics stability in wine. Polyphenols content and antioxidant activity changes during storage and wine ageing [2,14]. Furthermore, in some cases, additional treatments of wine can be necessary, such as a clarification process with fining agents that can change the content of phenolic compounds and antioxidant activity [15].

Membrane filtration includes four main processes: reverse osmosis (RO), nanofiltration (NF), ultrafiltration (UF) and microfiltration (MF) [16]. All of them are based on the application of selective membranes with different pore size (the smallest pores have RO membranes, followed by NF ones and so on). Membranes split the initial feed on retentate with increased solid concentration, and permeate that passes through the membrane [17]. The permeate flux is ensured with pressure application—the smaller the membrane pore size, the higher pressure is required. Nanofiltration membranes retain small molecules and ions that create high osmotic pressure on the membrane surface, and this requires the application of high working pressure (>2.0 MPa) [18]. However, the retention of molecules on the membrane surface leads to membrane fouling, concentration polarization and permeate flux decrease that can limit the use of nanofiltration membranes [19]. On the other hand, the retention of low molecular weight compounds makes the NF membranes applicable for wine concentration. The advantages of the nanofiltration process include high efficiency due to membrane selectivity and the ability to operate at room temperatures that minimize thermal degradation of the initial feed [17,20]. For concentration purposes, reverse osmosis and nanofiltration membranes could be applied due to small pore size and high selectivity. Compared to the reverse osmosis membranes, nanofiltration ones have slightly larger pore size that decreases the retention of bioactive compounds. However, at the same operating conditions, the permeate flux during nanofiltration is higher, the process duration time is shorter and membrane fouling is less severe than during the reverse osmosis process, which lowers the production cost [9]. The nanofiltration wine permeate contains mainly water and ethanol, but several low weight molecules can also pass through membranes, such as acetic and lactic acid, or some aroma compounds [18]. Therefore, the nanofiltration process can be used for wine or must concentration [21,22], partial dealcoholisation of wine [23,24], acetic acid removal [25,26], aroma and phenolic contents correction [9,18]. In addition, nanofiltration can be used for the fractionation and extraction of polyphenols from grape pomace [27,28]. Wine concentration by nanofiltration results in a wine retentate with decreased ethanol and water content that makes it applicable for low-alcohol wine production or excessive alcohol content removal [29]. Furthermore, wine retentate contains higher concentrations of bioactive compounds than the initial wine that improves its nutritional value and quality [22]. Such obtained retentate could be used as a drink or for further production of wine with a desired phenolic profile. The centre of interest of several previous studies included the influence of the nanofiltration process on phenolic compounds retention and antioxidant activity [9,22,23,30]. They all concluded that the nanofiltration process was applicable for wine concentration with moderate operation and investment costs.

The aim of this study was to investigate the change of phenolic profile, antioxidant activity and colour of conventional and ecological Cabernet Sauvignon red wine during concentration by nanofiltration at 2.5, 3.5, 4.5 and 5.5 MPa with and without cooling. In initial wines and NF retentates, the total polyphenol and flavonoid content, individual phenolic compounds and anthocyanins content, antioxidant activity and colour parameters were determined. The influence of the applied processing parameters and wine type on the mentioned compounds were monitored.

## 2. Materials and Methods

### 2.1. Reagents and Standards

In this study, the following reagents and standards were used: aluminium chloride, quercetin dihydrate, gallic acid monohydrate, potassium persulfate, Trolox, 2,2-azinobis(3-ethylbenzothiazoline sulfonic acid) (ABTS), 2,2-diphenyl-1-picrylhydrazil (DPPH), 2,4,6-tripyridyl-s-triazine (TPTZ) were obtained from Sigma-Aldrich (St. Lois, MO, USA); HPLC standards (gallic and caffeic acid, (+)-catechin hydrate, (−)-epicatechin, rutin hydrate, quercetin) were purchased from Sigma-Aldrich Chemie Gmbh (Steinheim, Germany), and malvidin 3-glucoside from Extrasynthese (Genay, France). Folin–Ciocalteu reagent, sodium nitrite, sodium carbonate, sodium hydroxide, potassium bisulphite, sodium acetate, potassium chloride and hydrochloric acid were purchased from Kemika (Zagreb, Croatia); sodium acetate trihydrate, ferric chloride hexahydrate, and ammonium acetate were purchased from Gram-Mol (Zagreb, Croatia); copper(II) chloride from Acros Organics (New Jersey, NJ, USA); HPLC grade methanol and neocuproine from Merck (Darmstadt, Germany) and phosphoric acid (HPLC grade) were obtained from Fluka (Buchs, Switzerland).

### 2.2. Conventional and Ecological Wine

Conventional and ecological Cabernet Sauvignon red wines (vintage 2018) were produced at cultivation area Zmajevac, Baranja vineyard, Croatia.

### 2.3. Nanofiltration Process

The operating conditions of the nanofiltration (NF) process of conventional and ecological wine were as follows: four pressures (2.5, 3.5, 4.5 and 5.5 MPa) and two temperature regimes (with and without cooling). For that purpose, laboratory plate-and-frame filter LabUnit M20 (De Danske Sukkerfabrikker, Nakskov, Denmark) was used. Six polyamide NF M20 membranes were inserted into the module. The maximum operating pressure for those membranes was 5.5 MPa, the maximum temperature was 50 °C and the pH range was between 3 and 10. The retention of MgSO_4_ measured on 2000 ppm, 0.9 MPa and 25 °C for those membranes was ≥99%. The surface of one membrane was 0.0289 m^2^. The initial feed volume was 3 L and the temperature was 15 °C. During the NF process, the permeate volume and retentate temperature were measured every four minutes. At the end of each experimental run, 1.3 L of retentate and 1.7 L of permeate was obtained. Retentates were diluted with distilled water to the initial wine volume before each analysis.

### 2.4. Calculation of Processing Parameters

The following formulas were used for the calculation of permeate flux (J) and volume reduction factor (VRF):

J = V_p_/(A × t), (1)

VRF = V_f_/V_r_, (2)

where V_p_ is permeate volume (L), A represents the surface of a membrane (m^2^) and t is the process duration (hours), V_f_ is the volume of the initial feed (L) and V_r_ is the volume of retentate (L).

### 2.5. Total Phenolic Compounds and Antioxidant Activity Determination

Total phenolic compounds in conventional and ecological wine and nanofiltration retentates were determined spectrophotometrically: total polyphenols content by the Folin–Ciocalteu method [31], total flavonoids content according to Kim et al. [32], and monomeric anthocyanins content (pH-differential method) and polymeric colour according to Giusti and Wrolstad [33]. The total polyphenols content was expressed as gallic acid equivalents (g GAE/L) and total flavonoids content as catechin equivalents (g CE/L). Samples were analysed in triplicates and the results were expressed as average values.

Antioxidant activity was determined spectrophotometrically according to four different methods: DPPH (2,2-diphenyl-1-picrylhydrazyl) [34], ABTS (2,2′-azinobis3-ethyl-benzothiazoline-6-sulfonic acid)) [35], FRAP (ferric reducing/antioxidant power assay) [36] and CUPRAC (cupric reducing antioxidant capacity) [37]. Three repetitions were made for each sample and the results were expressed as Trolox equivalents (μmol TE/100 mL).

### 2.6. High-Performance Liquid Chromatography (HPLC)

High-performance liquid chromatography (HPLC) was used to identify individual phenolic compounds and anthocyanins in wines and retentates. The HPLC system 1260 Infinity II (Agilent Technologies, Santa Clara, CA, USA) was equipped with Poroshell 120 EC-C18 column (4.6 × 100 mm, 2.7 μm), quaternary pump and diode array detector (DAD). Mobile phase A was 0.1% H_3_PO_4_, and mobile phase B was 100% methanol. For the determination of individual phenolics and hydroxycinnamic acids, the flavon-3-ols and flavonoids injection volume was 10 μL and the flow was set to 1 mL/min with the following gradient: 0 min 5% B, 3 min 30% B, 15 min 35% B, 22 min 37% B, 30 min 41% B, 32 min 45% B, 40 min 49% B, 45 min 80% B, 48 min 80% B, 50 min 5% B, 53 min 5% B. For anthocyanins determination, the injection volume was 20 μL and the gradient was as follows: 0–38 min, 3–65% B; 38–45 min, 65% B. For both methods, the UV/Vis spectra were recorded at wavelengths between 190 to 600 nm. The calibration curves in different concentration ranges were made for standards: 25–500 mg/L for gallic acid (r^2^ = 0.9986), (+)-catechin (r^2^ = 0.9997) and (−)-epicatechin (r^2^ = 0.9984), 0.25–10 mg/L for caffeic acid (r^2^ = 0.9989), rutin (r^2^ = 0.9989) and quercetin (r^2^ = 0.9995), 1–150 mg/L for malvidin 3-glucoside (r^2^ = 0.9994). Caftaric acid was tentatively identified through retention time, peak spectrum of authentic standards and literature data and the results were expressed through the caffeic acid calibration curve. In addition, quercetin derivative 1 and 2, and malvidin 3-glucoside derivative were tentatively identified using calibrations curves of quercetin and malvidin 3-glucoside. Two repetitions were made for each sample.

### 2.7. Measurement of Colour Parameters

In order to determine colour parameters in the CIELab system in initial wines and NF retentates, a chromometer CR-400 (Konica Minolta, Inc., Osaka, Japan) was used. In all the samples, the parameters L*, a*, b*, C* and °h were measured. Parameter L* represents lightness and ranges between 0 (black) and 100 (white); a* indicates redness if positive or greenness if negative; b* indicates yellowness if positive and blueness if negative; C* is the colour saturation and °h represents the hue angle [9,38]. The results were expressed as an average value of three repetitions. For colour difference determination, parameter ΔE* was calculated:

ΔE* = [(ΔL*)^2^ + (Δa*)^2^ + (Δb*)^2^]^1/2^ (3)

### 2.8. Statistical Analysis of Results

The results were expressed through the average value of repetitions with standard deviation. For statistical analysis, STATISTICA 13.1 (StatSoft Inc., Tulsa, OK, USA) software program was used, where analysis of variance (ANOVA), post-hoc Fisher’s least significant difference (LSD) test (*p* < 0.05) and principal component analysis (PCA) were applied. The correlation coefficient between results was calculated in MS Excel (Microsoft Office Professional, 2016).

## 3. Results

### 3.1. Nanofiltration Process

**Figure 1:**
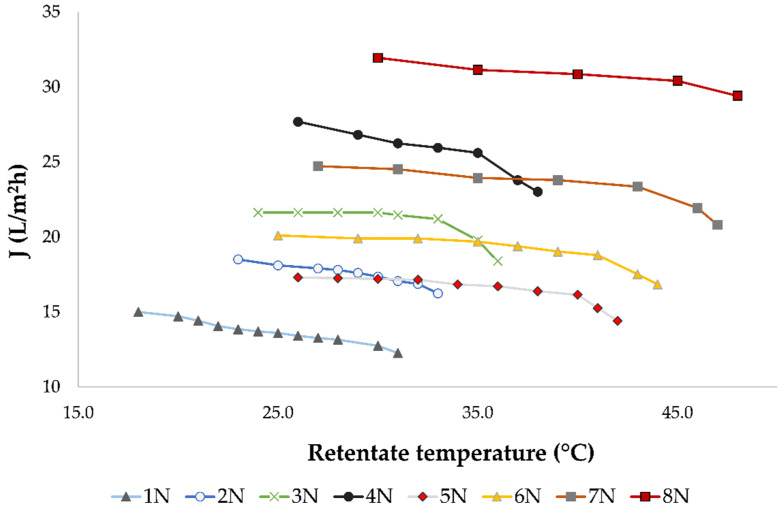
Influence of retentate temperature (°C) on permeate flux J (L/m^2^h) during nanofiltration of conventional and ecological Cabernet Sauvignon red wine at 2.5, 3.5, 4.5 and 5.5 MPa with and without cooling. Abbreviations: N—nanofiltration retentate; 1—2.5 MPa with cooling; 2—3.5 MPa with cooling; 3—4.5 MPa with cooling; 4—5.5 MPa with cooling; 5—2.5 MPa without cooling; 6—3.5 MPa without cooling; 7—4.5 MPa without cooling; 8—5.5 MPa without cooling.

The influence of processing parameters on permeate flux, bioactive compounds retention and membrane fouling during the nanofiltration (NF) of Cabernet Sauvignon red wine was explained in more detail in our previous studies [9,18]. In this study, during nanofiltration of conventional and ecological wine, similar results were obtained. Figure 1 illustrates the influence of retentate temperature under different operating conditions on permeate flux.

It has been observed that increased pressure and a lack of retentate cooling resulted in higher average permeate flux. The highest average permeate flux during the nanofiltration treatment of conventional and ecological wine was measured at 5.5 MPa without cooling (30.8 L/m^2^h), where the highest final retentate temperature was measured (48.0 °C). The cooling regime resulted in lower permeate fluxes comparing to the regime without cooling at the same applied pressure. It also resulted in a 10 to 11 °C lower final retentate temperature.

**Figure 2:**
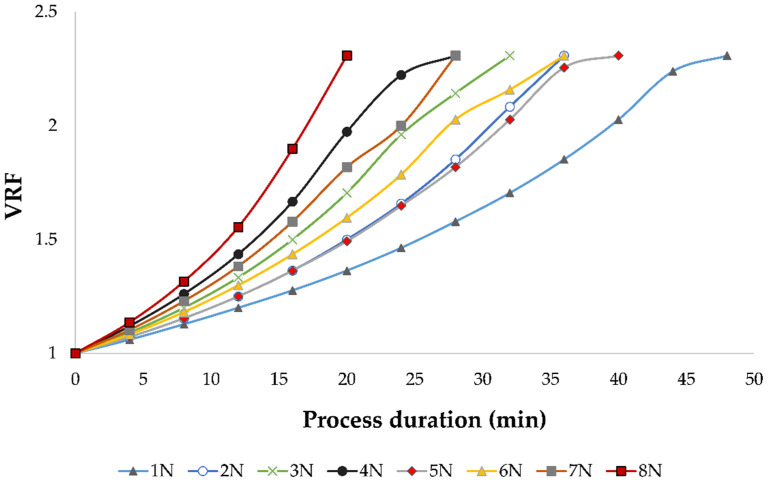
Influence of process duration (min) on volume reduction factor (VRF) during nanofiltration of conventional and ecological Cabernet Sauvignon red wine at 2.5, 3.5, 4.5 and 5.5 MPa with and without cooling. Abbreviations: N—nanofiltration retentate; 1—2.5 MPa with cooling; 2—3.5 MPa with cooling; 3—4.5 MPa with cooling; 4—5.5 MPa with cooling; 5—2.5 MPa without cooling; 6—3.5 MPa without cooling; 7—4.5 MPa without cooling; 8—5.5 MPa without cooling.

During the concentration process, a decline of permeate flux was observed regardless of the applied pressure and temperature, due to membrane fouling. This limited the nanofiltration process to the volume reduction factor (VRF) of 2.31. The VRF value increased as the retentate volume decreased. If the applied pressure was lower, the permeate flux was lower and more time was required to obtain the same VRF, especially when cooling was applied. This can be observed in Figure 2. The longest NF process was at 2.5 MPa with cooling, where it took 48 min to achieve the mentioned VRF. At 5.5 MPa without cooling, the high pressure and high permeate flux resulted in the desired retentate volume and VRF after only 20 min.

### 3.2. Total Phenolic Compounds Retention

The phenolic compounds content of the initial conventional and ecological Cabernet Sauvignon red wines and their NF retentates at different processing parameters are presented in Table 1 and Table 2. In each sample, the total polyphenols content (TPC), total flavonoids content (TFC), monomeric anthocyanins content (MAC) and polymeric colour (PC) were determined.

The TPC in initial conventional and ecological wine was 3.19 and 3.34 g/L, respectively. After the nanofiltration process, the total polyphenols contents were lower in all retentates (conventional and ecological wine) comparing to the initial wines. The retention of TPC increased with higher pressure and the highest contents were measured in conventional retentates obtained at 4.5 MPa and 5.5 MPa at both temperature regimes (2.40 to 2.49 g/L). The highest TPC among ecological wine retentates was measured at 5.5 MPa with and without cooling (2.84 g/L). There was no significant difference between two temperature regimes, with and without cooling, at the same transmembrane pressures. The lowest retention was observed at 2.5 MPa with and without cooling in both wine retentates. The total flavonoids contents also decreased after nanofiltration treatment comparing to the initial conventional (1.55 g/L) and ecological (1.64 g/L) red wines. The highest retention in conventional wine retentates was determined at 4.5 MPa and 5.5 MPa with cooling (1.31 g/L) and the lowest ones at 2.5 and 3.5 MPa at both temperature regimes without significant difference among contents (around 1.20 g/L). The regime without cooling resulted in slightly lower retention of TFC at 4.5 and 5.5 MPa (1.24 g/L) comparing to the cooling regime. In ecological wine retentates, the highest retention of TFC was observed at 4.5 and 5.5 MPa at both temperature regimes (1.48 g/L), and the lowest one at 2.5 MPa without cooling (1.15 g/L). The contents of MAC in the initial conventional and ecological wine were 151.41 and 103.83 mg/L, respectively. Their contents decreased after the NF process in both wine retentates, but the retention increased with the pressure increment. The highest contents were measured in retentates obtained at 5.5 MPa with cooling (124.12 g/L in conventional and 99.24 g/L in ecological wine retentates). Higher temperatures (regime without cooling) and lower pressure resulted in lower retention of MAC than during the cooling regime at higher pressures in both wine retentates. There was no significant change in polymeric colour in wine retentates obtained at cooling regime comparing to the initial conventional (61.50%) and ecological wines (68.82%). However, an increase of around 2% of PC was observed when cooling was not applied with no significant difference among applied pressures.

Comparing both wine retentates, it can be observed that the retention of total phenolic compounds in ecological wine retentates at cooling regime was higher than the retention of those compounds in conventional wine retentates. For comparison, the retention of TPC, TFC and MAC in ecological wine retentates at 5.5 MPa with cooling was 85.13%, 90.60% and 87.87%, and those values in conventional wine retentates were 77.95%, 83.72% and 81.98%, respectively. Similar trend was observed when cooling was not applied, except for TFC at 2.5 MPa, where the retention was higher in conventional wine retentate (76.82%) than in the ecological one (70.03%).

### 3.3. Individual Phenolic Compounds Retention

In conventional and ecological wine and NF retentates, gallic, caffeic, caftaric acid, (+)-catechin, (−)-epicatechin, rutin, quercetin and its two derivatives, malvidin 3-glucoside and its derivative were determined by high-performance liquid chromatography (HPLC) and the results are presented in Table 3 and Table 4.

The concentrations of individual phenolic compounds differed between initial conventional and ecological wine, although in both wines similar TPC and TFC values were determined (Table 1 and Table 2). Initial conventional wine contained higher concentrations of caffeic and caftaric acid, (+)-catechin and quercetin derivative 1 than the initial ecological wine. On the other hand, initial ecological wine contained higher concentrations of gallic acid, (−)-epicatechin, rutin, quercetin and quercetin derivative 2. The concentrations of malvidin 3-glucoside and its derivative were higher in initial conventional wine than in initial ecological wine, which corresponded to the higher content of MAC in initial conventional wine.

The results showed that the nanofiltration process of conventional and ecological wine resulted in a loss of individual phenolic compounds and anthocyanins. However, the retention of those compounds depended on applied processing parameters and type of wine. During the nanofiltration of conventional Cabernet Sauvignon wine, the highest retention of gallic, caffeic, caftaric acid, (+)-catechin, (−)-epicatechin, rutin, quercetin and its two derivatives, malvidin 3-glucoside and its derivative was observed at 5.5 MPa with cooling. When cooling was not applied, lower retention of those compounds was estimated, especially at 2.5 MPa, where the lowest concentrations were measured. Among conventional wine retentates obtained at the regime without cooling, the highest retention was achieved at 4.5 MPa. Applied NF membranes retained 100% of (−)-epicatechin in conventional wine retentate obtained at 5.5 MPa with cooling. In the same retentate, the retention of caftaric acid, quercetin derivative 2, malvidin 3-glucoside and its derivative was higher than 90%. The lowest retention in the mentioned retentate was measured for quercetin (21.19%). However, rutin concentrations were slightly increased during nanofiltration process of conventional wine, compared to the initial value (0.95 mg/L). The exception was the conventional wine retentate obtained at 2.5 MPa where the concentration of rutin did not significantly change compared to the concentration in initial conventional wine.

In ecological wine retentates, the retention of phenolic compounds and anthocyanins did not always follow the same trend. The lowest retention of all compounds was mostly observed at 2.5 MPa without cooling. The retention of gallic acid, (+)-catechin and (−)-epicatechin was the highest at 5.5 MPa with cooling (68.26%, 86.89% and 76.30%, respectively). This indicated that high pressure and retentate cooling favoured the retention of mentioned compounds during nanofiltration of ecological wine. Processing parameters affected the retention of other compounds differently. For example, the highest concentrations of caffeic acid and malvidin 3-glucoside derivative (72.38% and 65.47% of the initial value, respectively) were measured in ecological wine retentates obtained at 4.5 and 5.5 MPa without cooling and there was no significant difference between those pressures. The highest retention of rutin and quercetin derivative 1 (84.28% and 63.64% of the concentration in initial ecological wine, respectively) was achieved at 3.5, 4.5 and 5.5 MPa without cooling, with no significant difference among values. The concentration of quercetin derivative 2 in initial ecological wine was 1.20 mg/L, and the lowest retention of this compound was observed at 2.5 MPa without cooling (0.89 mg/L). Among the rest of the obtained ecological wine retentates, no significant difference in quercetin derivative 2 values was observed (0.99 mg/L). The highest retention of malvidin 3-glucoside was achieved at 3.5 and 4.5 MPa without cooling (around 80.52% of initial value), and the highest retention of malvidin 3-glucoside derivative was observed at 4.5 and 5.5 MPa without cooling (65.47% of initial concentration). Initial ecological wine contained 3.57 mg/L of quercetin, but it was not detected in any ecological wine retentate.

**Figure 3:**
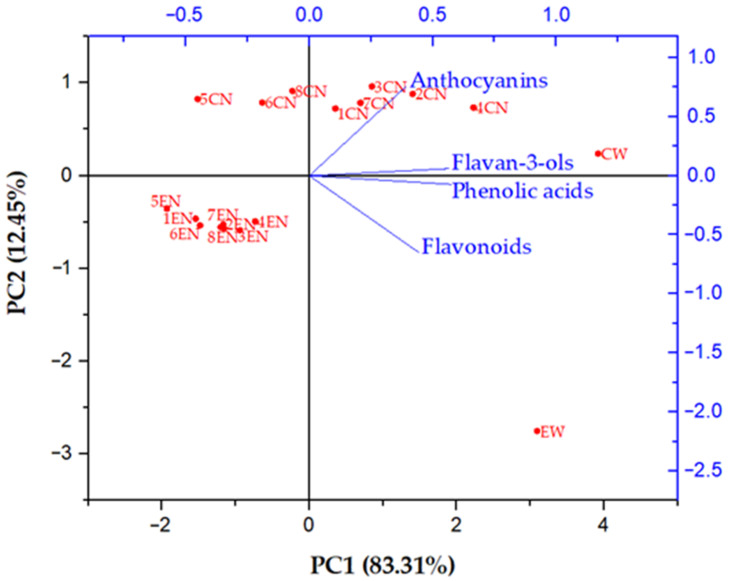
Principal component analysis (PCA) biplot of phenolic profiles obtained by HPLC of initial wines and nanofiltration retentates. Abbreviations: CW—initial conventional wine; EW—initial ecological wine; CN—nanofiltration retentate of conventional wine; EN—nanofiltration retentate of ecological wine; 1—2.5 MPa with cooling; 2—3.5 MPa with cooling; 3—4.5 MPa with cooling; 4—5.5 MPa with cooling; 5—2.5 MPa without cooling; 6—3.5 MPa without cooling; 7—4.5 MPa without cooling; 8—5.5 MPa without cooling.

In order to compare the phenolic profiles of conventional and ecological Cabernet Sauvignon red wine and their nanofiltration retentates obtained under different operating conditions, principal component analysis (PCA) was conducted (Figure 3). For a better display, phenolic compounds were divided into four groups: phenolic acids (gallic, caffeic and caftaric acid), flavan-3-ols (catechin and epicatechin), flavonoids (rutin, quercetin, quercetin derivatives 1 and 2) and anthocyanins (malvidin 3-glucoside and its derivative). Principal component 1 (PC1) accounted for 83.31% and principal component 2 (PC2) 12.45% of total variance. PC1 separates the samples according to the applied processing parameters, and PC2 splits them on the conventionally (positive side) and ecologically (negative side) produced ones. It can be observed that the phenolic profiles of initial conventional and ecological wine were different due to different concentrations of individual phenolic compounds. After the nanofiltration process, the phenolic profile changed in all retentates. Ecological wine retentates were all clustered at the negative sides of PC1 and PC2, with very small differences regarding the applied processing parameters. On the other hand, significant differences were visible among conventional wine retentates obtained at different pressures and temperature regimes. The conventional wine retentates obtained at 2.5, 3.5 and 5.5 MPa with cooling were located on the negative side and the rest of them were on the positive side of PC1.

All conventional wine retentates were located on the positive side of PC2. It can be observed that the retention of phenolic compounds during the nanofiltration process was higher in conventional wine retentates than in the ecological ones, especially in the retentate obtained at 5.5 MPa with cooling, where the slightest change of phenolic profile occurred, comparing to the initial conventional wine. This retentate was closest to the initial wine on the PCA biplot. Nanofiltration treatment of ecological wine resulted in a significant change of phenolic profile regardless of the applied pressure and temperature, in comparison to the initial ecological wine.

### 3.4. Antioxidant Activity

Antioxidant activities of initial wines and NF retentates at 2.5, 3.5, 4.5 and 5.5 MPa with and without cooling were determined by four methods (DPPH, ABTS, FRAP and CUPRAC) and the results are presented in Table 5 and Table 6.

Antioxidant activities of both initial wines were similar. In initial conventional wine, DPPH, ABTS, FRAP and CUPRAC values were 14.92, 35.18, 3.04 and 174.77 μmol/100 mL, and in initial ecological wine, these values were 14.77, 33.46, 3.10 and 170.85 μmol/100 mL, respectively. All four antioxidant assays resulted in different antioxidant activities due to different mechanisms for antioxidant activity determination. Nevertheless, the results of the DPPH, ABTS, FRAP and CUPRAC methods showed that all values decreased after nanofiltration treatment of both wines, and the antioxidant activities depended on applied processing parameters and wine type. Among NF retentates, the highest antioxidant activities determined by DPPH and CUPRAC were measured at 5.5 MPa with cooling (9.87 and 158.23 μmol/100 mL in conventional wine retentate and 11.15 and 149.84 μmol/100 mL in the ecological one, respectively). Transmembrane pressures of 4.5 and 5.5 MPa at regime with cooling resulted in the highest antioxidant activities determined by ABTS in both wine retentates (25.77 μmol/100 mL in conventional and 31.50 μmol/100 mL in ecological wine retentate), with no significant difference between those two pressures. After the nanofiltration process, antioxidant activities determined by FRAP were the highest for retentates obtained by 5.5 MPa with and without cooling in conventional (2.62 μmol/100 mL) and ecological (2.66 μmol/100 mL) ones, with no significant difference between two temperature regimes.

In comparison to the initial wines, antioxidant activities in NF retentates decreased by 5 to 80%, depending on the operating conditions and initial wine composition. The smallest decrease of antioxidant activity was evaluated by the ABTS method in ecological wine retentate at 4.5 and 5.5 MPa with cooling, where the antioxidant activity decreased only by 5.85%, compared to the initial value. In conventional wine retentate at the same operating conditions, a higher decrease of antioxidant activity, according to the ABTS method, was observed (26.75% of initial value). Antioxidant activities determined by the FRAP and CUPRAC method decreased by 9 to 40% after nanofiltration process of conventional and ecological wine. This depended on the applied processing parameters (higher pressure and lower temperature resulted in higher antioxidant activities). The highest decrease of antioxidant activity was evaluated by DPPH method in both wine retentates at 2.5 MPa without cooling where the DPPH values were 79.83% (conventional wine retentate) and 60.95% (ecological wine retentate) lower than in the corresponding initial wine.

The decrease in antioxidant activities after nanofiltration treatment of conventional and ecological wine corresponded to the decrease in total polyphenol and flavonoid content and monomeric anthocyanins. This is presented in Table 7, where correlation coefficients (*r*) between TPC, TFC, MAC and antioxidant activities (DPPH, ABTS, FRAP and CUPRAC) in initial conventional and ecological wine and their NF retentates were calculated.

The correlation coefficient represents the linear relationship between two variables, and it ranges from −1 (perfect negative linear relationship) to +1 (perfect positive linear relationship). The 0 indicates no linear relationship [39]. It can be observed from Table 7 that all values ranged from 0.759 to 0.983. This indicated a strong positive linear relationship between TPC, TFC, MAC values and antioxidant activities determined by DPPH, ABTS, FRAP and CUPRAC in initial conventional and ecological wine and their NF retentates. The lowest correlation coefficient was calculated for relationships TPC/CUPRAC and TFC/CUPRAC in conventional wine retentates (0.769 and 0.759, respectively), followed by a correlation coefficient of 0.787 between MAC and ABTS in ecological wine retentates. The rest of the correlation coefficient values were higher than 0.8. The strong positive linear relationship between phenolic compounds and antioxidant activities indicated that a higher retention of total phenolic compounds during the nanofiltration process of conventional and ecological wine resulted in higher antioxidant activities in retentates and vice versa. Higher pressure and retentate cooling were more favourable for TPC, TFC and MAC retention, and this corresponded to the higher antioxidant activities in retentates obtained at the same processing parameters.

### 3.5. Colour Parameters

The colour parameters (L*, a*, b*, C*, °h and ΔE*) of initial conventional and ecological wine and their NF retentates were determined and the results are presented in Table 8 and Table 9. It can be observed that in both initial wines the L* value was 19.70 and it slightly increased after nanofiltration process with no significant difference among retentates obtained at different operating conditions. Slightly higher L* values were measured in ecological wine retentates (average value was 20.31) than in the conventional ones (average value was 19.99). The initial a* value did not change during the NF process with cooling, and it slightly decreased when cooling was not applied in both wine retentates. There was no significant change of b* value in all NF retentates comparing to the initial wines. The hue angle in initial ecological wine was 33.54 and it slightly decreased in NF retentates with no significant difference among them regarding the applied pressure and temperature. In initial conventional wine, the °h value was 35.80; it decreased after the NF process but slightly higher °h values were measured in retentates obtained at 4.5 and 5.5 MPa with and without cooling than in the ones obtained at lower pressures. The C* value was higher in NF retentates than in initial conventional wine, with the highest value measured at 4.5 and 5.5 MPa without cooling. The highest C* values among ecological wine retentates were measured at 4.5 and 5.5 MPa with and without cooling. The ΔE* value was lower than 1.00 in all retentates but slightly higher in ecological wine retentates than in the conventional ones. Regarding the applied pressure and temperature, a similar trend was observed in both wine retentates: slightly lower values of ΔE* were obtained at pressures 2.5, 3.5 and 4.5 MPa with cooling, and there was no significant difference among other retentates.

## 4. Discussion

Nanofiltration (NF) is a pressure-driven membrane separation technique that has applications in the wine industry for ethanol or acetic acid removal, aroma, phenolic or sugar correction, and others [9]. The composition of wine retentates obtained by nanofiltration depended on the applied processing parameters, mainly pressure and temperature. In this study, the influence of four different pressures (2.5, 3.5, 4.5 and 5.5 MPa) and two temperature regimes (with and without cooling) was monitored during the nanofiltration of conventional and ecological Cabernet Sauvignon red wine. Higher pressure resulted in higher permeate flux and higher retentate temperature, which was consistent with previous studies [9,18,19,23,27,40]. Pressure and temperature increase resulted in a higher permeate flux and shorter NF process, but also in a higher risk of thermal degradation of retentate components, especially when cooling was not applied. Higher permeate flux at high pressures was a result of increased interactions between water and hydrophilic part of the membrane that resulted in higher permeability of water than other compounds [41]. This resulted in faster wine concentration, so the targeted retentate volume and volume reduction factor (VRF) was achieved sooner. Furthermore, high pressure led to faster membrane fouling, concentration polarization and cake formation that resulted in higher bioactive compounds retention and permeate flux decline [40,42,43]. Salgado et al. [44] reported that high molecular weight compounds, such as certain polyphenols, polysaccharides or proteins, were accumulated on the membrane surface and they formed a pseudo membrane that increased the retention of bioactive compounds, such as anthocyanins, and decreased the permeate flux. Temperature increase resulted in higher permeate flux comparing to the one achieved at the same pressure with cooling due to lower viscosity of the retentate [23]. In several previous studies, similar results were obtained regarding the pressure and temperature influence on permeate flux during the concentration of wine [22,23], grape juice [45] or chokeberry juice [19].

Furthermore, the retention of bioactive compounds depended on the applied operating conditions, but it also depended on the chemical properties of compounds, membrane characteristics and the interactions between retentate components and the membrane surface [18]. The molecular weight cut-off (MWCO) of a nanofiltration membrane is usually up to 1000 Da or g/mol [46], meaning that it permeated the compounds with molecular weight lower than the MWCO value, including water (18.02 g/mol) and ethanol (46.07 g/mol). The accumulation of organic molecules, colloids or salts on the membrane surface resulted in membrane fouling [47]. Each compound contributed to the membrane fouling, and the retention or permeability of compounds depended on its molecular weight and chemical properties (electrical charge and polarity, ability to interact with other compounds) [48]. Compounds with the same hydrophobic character as the membrane or a part of a membrane would be attracted to the membrane and its permeability would increase. Hence, nonpolar membranes showed higher rejection towards polar compounds and vice versa [49].

In this study, the influence of processing parameters and wine type (conventionally and ecologically produced one) on phenolic compounds during nanofiltration was investigated. In conventional and ecological Cabernet Sauvignon red wine, gallic, caftaric and caffeic acid, rutin, quercetin and its derivatives, (+)-catechin, (−)-epicatechin, malvidin 3-glucoside and its derivative were determined, which are characteristic for the analysed wines [9,50,51]. Malvidin 3-glucoside and its derivatives are usually the most abundant anthocyanins in red wines and are mainly responsible for their colour [52]. The results showed that the nanofiltration processing parameters did not affect each phenolic compound the same way and that influence differed between conventional and ecological wine. The principal component analysis (PCA) showed that the initial phenolic profile of conventional wine was different from the ecological one due to different concentrations of individual phenolic compounds. Higher pressure (5.5 MPa) and retentate cooling resulted in high retention of individual phenolic compounds in conventional wine retentates. Opposite processing parameters resulted in a significant change of phenolic profile of conventional wine. After nanofiltration, the phenolics content of ecological wine changed significantly, but only slight differences were noticed among ecological wine NF retentates regarding applied pressure and temperature. According to the obtained phenolic profile, in conventional wine retentates, during the nanofiltration process at higher pressures and lower temperatures, higher retention of individual compounds was observed than in ecological wine retentates. It can be concluded that different chemical compositions of the initial feed, specifically the wine matrix, can influence the retention of phenolic compounds. López-Muňoz et al. [53] stated that the pH affected the phenolics retention due to change of the membrane active layer and phenolic compounds dissociation. If the pH is closer to the isoelectric point of a membrane, the retention of bioactive compounds will decrease [54]. The isoelectric point of polyamide membranes is usually at pH 3.5 to 4.0 [55], and the pH values of conventional and ecological wine used in this study were 3.92 and 3.75, respectively. Furthermore, Arsuaga et al. [56] reported that the retention of the phenolic compounds depended on their adsorption on nanofiltration membranes. The adsorption occurred due to hydrophobic interactions between the phenolics and membrane that can also lead to higher membrane fouling. Each individual phenolic compound affected the membrane fouling differently, and it depended on its chemical properties, molecular refractive index, acidity coefficient and membrane characteristics [48].

In this study, the total polyphenols content (TPC), total flavonoids content (TFC) and monomeric anthocyanins content (MAC) were determined in all samples. Generally, higher pressure favoured the retention of the mentioned compounds. The retention of anthocyanins increased when temperatures were lower. Higher temperatures cause higher permeability or thermal degradation of anthocyanins and that could lead to an increase in polymeric colour [19]. Polymeric colour represents the colour derived from polymerized material formed by the conversion of anthocyanins into undesirable colourless or brown compounds [57]. In this study, the polymeric colour increase was observed when cooling was not applied, but pressure change had no significant influence on it. Banvolgyi et al. [23] studied the influence of temperature on resveratrol and anthocyanins content in red wine during nanofiltration. They stated that lower temperatures (20 °C) resulted in higher retention of mentioned compounds.

The colour of initial wines and NF retentates was evaluated by CIELab system. The results showed that only slight differences were observed in both conventional and ecological wine retentates. To establish the differences among retentates and initial wines, the ΔE* value was calculated. In all retentates, this value was lower than 1. This means that the human eye would not be able to distinguish the colour change [58]. Pressure and temperature had no significant influence on colour change during nanofiltration, except for the a* value (redness), which slightly decreased at higher temperatures. These results are consistent with our previous study where it was reported that the retentate colour did not significantly differ from the initial wine colour [9].

## 5. Conclusions

The results of the investigation of phenolic compounds and the antioxidant activity of the obtained retentates showed that nanofiltration membranes could be used for the concentration of conventional and ecological Cabernet Sauvignon red wine. The influence of different pressures and temperature regimes on phenolic compounds retention was observed. In both wine retentates, a slight loss of phenolic compounds was observed after the nanofiltration process, but the high pressure and retentate cooling favoured the retention of total phenolic and flavonoid compounds and monomeric anthocyanins. A similar trend was observed for the antioxidant activity in both wines. The retention of individual phenolic compounds depended on the processing parameters, wine type (conventional or ecological), chemical properties of each compound and membrane characteristics. Conventional and ecological wine showed different behaviour during nanofiltration and different retention of individual phenolic compounds were observed between both wine retentates at the same operating conditions.

## Figures and Tables

**Table 1:** Total phenolic compounds content of initial conventional Cabernet Sauvignon wine and retentates obtained by nanofiltration at 2.5, 3.5, 4.5 and 5.5 MPa with and without cooling.

Sample	TPC (g GAE/L)	TFC (g CE/L)	MAC (mg CGE/L)	PC (%)
CW	3.19 ± 0.06 ^d^	1.55 ± 0.04 ^d^	151.41 ± 0.49 ^g^	61.50 ± 0.22 ^a^
1CN	2.14 ± 0.03 ^a^	1.18 ± 0.03 ^a^	99.15 ± 0.36 ^c^	61.76 ± 0.16 ^a^
2CN	2.26 ± 0.06 ^b^	1.17 ± 0.03 ^a^	101.49 ± 0.87 ^d^	61.94 ± 0.55 ^a^
3CN	2.40 ± 0.04 ^b,c^	1.31 ± 0.03 ^c^	108.79 ± 0.66 ^e^	61.89 ± 0.21 ^a^
4CN	2.49 ± 0.06 ^c^	1.30 ± 0.02 ^c^	124.12 ± 0.64 ^f^	61.52 ± 0.38 ^a^
5CN	2.08 ± 0.04 ^a^	1.19 ± 0.03 ^a^	78.28 ± 0.22 ^a^	63.63 ± 0.29 ^b^
6CN	2.32 ± 0.04 ^b^	1.20 ± 0.01 ^a^	96.80 ± 0.09 ^b^	63.64 ± 0.11 ^b^
7CN	2.42 ± 0.01 ^c^	1.24 ± 0.01 ^b^	99.95 ± 0.86 ^c^	63.67 ± 0.14 ^b^
8CN	2.46 ± 0.03 ^c^	1.24 ± 0.01 ^b^	109.99 ± 0.56 ^e^	63.95 ± 0.32 ^b^

Different superscript letters indicate significant differences among samples within the column (*p* < 0.05; ANOVA, Fisher’s LSD test). Abbreviations: CW—initial conventional wine; CN—nanofiltration retentate of conventional wine; 1—2.5 MPa with cooling; 2—3.5 MPa with cooling; 3—4.5 MPa with cooling; 4—5.5 MPa with cooling; 5—2.5 MPa without cooling; 6—3.5 MPa without cooling; 7—4.5 MPa without cooling; 8—5.5 MPa without cooling; TPC—total polyphenols content; TFC—total flavonoids content; MAC—monomeric anthocyanins content; PC—polymeric colour.

**Table 2:** Total phenolic compounds content of initial ecological Cabernet Sauvignon wine and retentates obtained by nanofiltration at 2.5, 3.5, 4.5 and 5.5 MPa with cooling and without cooling.

Sample	TPC (g GAE/L)	TFC (g CE/L)	MAC (mg CGE/L)	PC (%)
EW	3.34 ± 0.06 ^e^	1.64 ± 0.02 ^d^	103.83 ± 0.72 ^f^	68.62 ± 0.97 ^a^
1EN	2.35 ± 0.03 ^a^	1.30 ± 0.01 ^b^	84.95 ± 0.96 ^b^	68.63 ± 0.35 ^a^
2EN	2.45 ± 0.03 ^b^	1.33 ± 0.02 ^b^	87.54 ± 0.69 ^c^	68.62 ± 0.36 ^a^
3EN	2.62 ± 0.10 ^c^	1.50 ± 0.02 ^c^	88.62 ± 0.86 ^c^	68.20 ± 0.43 ^a^
4EN	2.84 ± 0.05 ^d^	1.48 ± 0.02 ^c^	99.24 ± 0.45 ^e^	68.82 ± 0.31 ^a^
5EN	2.28 ± 0.04 ^a^	1.15 ± 0.02 ^a^	78.16 ± 0.86 ^a^	70.93 ± 0.13 ^b^
6EN	2.45 ± 0.01 ^b^	1.30 ± 0.02 ^b^	85.11 ± 0.92 ^b^	70.85 ± 0.18 ^b^
7EN	2.62 ± 0.05 ^c^	1.49 ± 0.03 ^c^	84.60 ± 0.95 ^b^	70.87 ± 0.24 ^b^
8EN	2.74 ± 0.05 ^d^	1.45 ± 0.05 ^c^	91.39 ± 0.29 ^d^	70.99 ± 0.38 ^b^

Different superscript letters indicate significant differences among samples within the column (*p* < 0.05; ANOVA, Fisher’s LSD test). Abbreviations: EW—initial ecological wine; EN—nanofiltration retentate of ecological wine; 1—2.5 MPa with cooling; 2—3.5 MPa with cooling; 3—4.5 MPa with cooling; 4—5.5 MPa with cooling; 5—2.5 MPa without cooling; 6—3.5 MPa without cooling; 7—4.5 MPa without cooling; 8—5.5 MPa without cooling; TPC—total polyphenols content; TFC—total flavonoids content; MAC—monomeric anthocyanins content; PC—polymeric colour.

**Table 3:** Concentration (mg/L) of individual phenolic compounds and anthocyanins in the initial conventional Cabernet Sauvignon wine and retentates obtained by nanofiltration at 2.5, 3.5, 4.5 and 5.5 MPa with cooling and without cooling.

**Sample**	**Gallic Acid**	**Caffeic Acid**	**Caftaric Acid**	**(+)-Catechin**	(−)-Epicatechin	Rutin	Quercetin	DQ1	DQ2	Malvidin 3-Glucoside	DM3-g
CW	42.22 ± 0.65 ^g^	2.71 ± 0.01 ^f^	11.18 ± 0.11 ^f^	88.71 ± 0.60 ^i^	34.63 ± 0.16 ^g^	0.95 ± 0.02 ^a^	1.18 ± 0.01 ^e^	2.02 ± 0.04 ^f^	1.11 ± 0.01 ^f^	38.57 ± 0.01 ^f^	8.27 ± 0.01 ^f^
1CN	26.41 ± 0.13 ^d^	1.76 ± 0.01 ^c^	9.23 ± 0.06 ^c^	65.94 ± 0.09 ^e^	31.52 ± 0.29 ^d^	1.06 ± 0.01 ^b^	0.18 ± 0.01 ^c^	0.96 ± 0.02 ^b^	0.86 ± 0.01 ^b^	28.23 ± 0.29 ^b^	6.11 ± 0.02 ^b^
2CN	29.18 ± 0.34 ^e^	1.93 ± 0.02 ^d^	9.95 ± 0.12 ^d^	76.79 ± 0.44 ^g^	34.02 ± 0.08 ^f^	1.15 ± 0.02 ^c^	0.21 ± 0.01 ^c^	1.11 ± 0.02 ^c^	0.93 ± 0.02 ^c^	32.71 ± 0.07 ^d^	6.86 ± 0.13 ^c^
3CN	28.85 ± 0.07 ^e^	1.88 ± 0.05 ^d^	9.41 ± 0.22 ^c,d^	70.12 ± 0.34 ^f^	33.02 ± 0.19 ^e^	1.03 ± 0.01 ^b^	0.17 ± 0.03 ^c^	0.95 ± 0.03 ^b^	0.85 ± 0.01 ^b^	31.18 ± 0.99 ^d^	6.56 ± 0.15 ^c^
4CN	32.16 ± 0.14 ^f^	2.15 ± 0.01 ^e^	10.96 ± 0.09 ^e^	79.33 ± 0.55 ^h^	34.65 ± 0.19 ^g^	1.30 ± 0.01 ^e^	0.25 ± 0.01 ^d^	1.39 ± 0.01 ^e^	1.05 ± 0.01 ^e^	35.16 ± 0.52 ^e^	7.51 ± 0.17 ^e^
5CN	20.68 ± 0.08 ^a^	1.42 ± 0.03 ^a^	7.46 ± 0.01 ^a^	45.35 ± 0.14 ^a^	26.03 ± 0.07 ^a^	0.91 ± 0.02 ^a^	0.08 ± 0.01 ^a^	0.77 ± 0.01 ^a^	0.72 ± 0.01 ^a^	25.86 ± 0.17 ^a^	5.30 ± 0.02 ^a^
6CN	22.88 ± 0.01 ^b^	1.67 ± 0.02 ^b^	8.35 ± 0.02 ^b^	51.43 ± 0.50 ^b^	28.89 ± 0.15 ^b^	1.06 ± 0.02 ^b^	0.10 ± 0.02 ^a,b^	0.98 ± 0.01 ^b^	0.83 ± 0.01 ^b^	28.80 ± 0.13 ^b^	6.03 ± 0.05 ^b^
7CN	26.58 ± 0.09 ^d^	1.97 ± 0.03 ^d^	10.12 ± 0.09 ^d^	61.29 ± 0.10 ^d^	33.32 ± 0.05 ^e^	1.21 ± 0.01 ^d^	0.13 ± 0.01 ^b^	1.22 ± 0.01 ^d^	0.98 ± 0.01 ^d^	32.86 ± 0.21 ^d^	7.04 ± 0.05 ^d^
8CN	24.85 ± 0.25 ^c^	1.78 ± 0.02 ^c^	9.07 ± 0.04 ^c^	56.27 ± 0.16 ^c^	30.82 ± 0.09 ^c^	1.00 ± 0.03 ^b^	0.09 ± 0.02 ^a,b^	0.99 ± 0.01 ^b^	0.82 ± 0.01 ^b^	29.20 ± 0.02 ^c^	6.12 ± 0.01 ^b^

Significant differences (*p* < 0.05) between samples are indicated by different superscript letters within the column (ANOVA, Fisher’s LSD test). Abbreviations: CW—initial conventional wine; CN—nanofiltration retentate of conventional wine; 1—2.5 MPa with cooling; 2—3.5 MPa with cooling; 3—4.5 MPa with cooling; 4—5.5 MPa with cooling; 5—2.5 MPa without cooling; 6—3.5 MPa without cooling; 7—4.5 MPa without cooling; 8—5.5 MPa without cooling; DQ1 and DQ2—quercetin derivative 1 and quercetin derivative 2; DM3-g—malvidin 3-glucoside derivative.

**Table 4:** Concentration (mg/L) of individual phenolic compounds and anthocyanins in the initial ecological Cabernet Sauvignon wine and retentates obtained by nanofiltration at 2.5, 3.5, 4.5 and 5.5 MPa with cooling and without cooling.

**Sample**	**Gallic Acid**	**Caffeic Acid**	**Caftaric Acid**	**(+)-Catechin**	(−)-Epicatechin	Rutin	Quercetin	DQ1	DQ2	Malvidin 3-Glucoside	DM3-g
EW	43.95 ± 0.60 ^g^	2.10 ± 0.01 ^e^	4.05 ± 0.01 ^d^	42.18 ± 0.34 ^h^	69.80 ± 1.61 ^f^	1.59 ± 0.01 ^d^	3.57 ± 0.04 ^a^	1.43 ± 0.01 ^d^	1.20 ± 0.01 ^f^	16.12 ± 0.10 ^e^	3.07 ± 0.01 ^c^
1EN	26.08 ± 0.12 ^b,c^	1.36 ± 0.01 ^b^	3.42 ± 0.04 ^b^	30.01 ± 0.02 ^c^	48.31 ± 0.19 ^b^	1.28 ± 0.02 ^b^	^-^	0.70 ± 0.01 ^a^	0.96 ± 0.02 ^b^	11.77 ± 0.07 ^a^	1.83 ± 0.01 ^a^
2EN	28.02 ± 0.56 ^d^	1.46 ± 0.01 ^c^	3.61 ± 0.01 ^c^	31.64 ± 0.12 ^d^	51.36 ± 0.85 ^d^	1.31 ± 0.02 ^b^	^-^	0.74 ± 0.03 ^a^	0.99 ± 0.02 ^b^	11.68 ± 0.05 ^a^	1.82 ± 0.02 ^a^
3EN	29.68 ± 0.03 ^e^	1.49 ± 0.02 ^c,d^	3.63 ± 0.03 ^c^	32.00 ± 0.10 ^e^	53.22 ± 0.24 ^e^	1.29 ± 0.01 ^b^	^-^	0.81 ± 0.01 ^b^	0.98 ± 0.02 ^b^	11.64 ± 0.09 ^a^	1.83 ± 0.07 ^a^
4EN	30.00 ± 0.01 ^f^	1.44 ± 0.03 ^c^	3.51 ± 0.12 ^b,c^	36.65 ± 0.06 ^g^	53.26 ± 0.06 ^e^	1.29 ± 0.01 ^b^	^-^	0.82 ± 0.01 ^b^	0.96 ± 0.03 ^b^	12.39 ± 0.01 ^c^	1.86 ± 0.06 ^a^
5EN	23.59 ± 0.04 ^a^	1.25 ± 0.02 ^a^	3.18 ± 0.01 ^a^	28.47 ± 0.01 ^a^	46.12 ± 0.88 ^a^	1.20 ± 0.01 ^a^	^-^	0.71 ± 0.01 ^a^	0.89 ± 0.01 ^a^	12.19 ± 0.03 ^b^	1.82 ± 0.05 ^a^
6EN	25.56 ± 0.56 ^b^	1.34 ± 0.01 ^b^	3.37 ± 0.03 ^b^	29.68 ± 0.06 ^b^	47.82 ± 0.31 ^b^	1.33 ± 0.01 ^c^	^-^	0.90 ± 0.01 ^c^	0.99 ± 0.01 ^b^	12.92 ± 0.17 ^d^	1.90 ± 0.11 ^a,b^
7EN	26.68 ± 0.48 ^c^	1.52 ± 0.01 ^d^	3.61 ± 0.05 ^c^	32.22 ± 0.27 ^e^	48.95 ± 0.29 ^c^	1.34 ± 0.01 ^c^	^-^	0.91 ± 0.01 ^c^	1.02 ± 0.02 ^b^	12.98 ± 0.10 ^d^	2.01 ± 0.04 ^b^
8EN	27.17 ± 0.01 ^c^	1.52 ± 0.01 ^d^	3.59 ± 0.02 ^c^	32.87 ± 0.05 ^f^	49.28 ± 0.17 ^c^	1.33 ± 0.01 ^c^	^-^	0.90 ± 0.01 ^c^	1.01 ± 0.02 ^b^	12.51 ± 0.11 ^c^	2.01 ± 0.05 ^b^

Significant differences (*p* < 0.05) between samples are indicated by different superscript letters within the column (ANOVA, Fisher’s LSD test). Abbreviations: EW—initial ecological wine; EN—nanofiltration retentate of ecological wine; 1—2.5 MPa with cooling; 2—3.5 MPa with cooling; 3—4.5 MPa with cooling; 4—5.5 MPa with cooling; 5—2.5 MPa without cooling; 6—3.5 MPa without cooling; 7—4.5 MPa without cooling; 8—5.5 MPa without cooling; DQ1 and DQ2—quercetin derivative 1 and quercetin derivative 2; DM3-g—malvidin 3-glucoside derivative.

**Table 5:** Antioxidant activity determined by DPPH, ABTS, FRAP and CUPRAC of initial conventional Cabernet Sauvignon wine and retentates obtained by nanofiltration at 2.5, 3.5, 4.5 and 5.5 MPa with cooling and without cooling.

Sample	DPPH (µmol TE/100 mL)	ABTS (µmol TE/100 mL)	FRAP (µmol TE/100 mL)	CUPRAC (µmol TE/100 mL)
CW	14.92 ± 0.97 ^f^	35.18 ± 0.15 ^g^	3.04 ± 0.15 ^e^	174.77 ± 1.07 ^f^
1CN	3.74 ± 0.29 ^a,b^	20.32 ± 0.26 ^b^	2.28 ± 0.03 ^b^	136.06 ± 1.32 ^b^
2CN	3.77 ± 0.38 ^a,b^	24.65 ± 0.17 ^e^	2.30 ± 0.03 ^b^	149.85 ± 1.50 ^d^
3CN	8.89 ± 0.47 ^d^	25.68 ± 0.10 ^f^	2.47 ± 0.05 ^c^	151.76 ± 1.50 ^d^
4CN	9.87 ± 0.16 ^e^	25.77 ± 0.09 ^f^	2.62 ± 0.04 ^d^	158.23 ± 0.79 ^e^
5CN	3.01 ± 0.44 ^a^	16.43 ± 0.41 ^a^	2.09 ± 0.07 ^a^	116.44 ± 0.24 ^a^
6CN	4.05 ± 0.16 ^b^	22.15 ± 0.32 ^c^	2.24 ± 0.03 ^b^	117.31 ± 1.66 ^a^
7CN	6.45 ± 0.32 ^c^	23.13 ± 0.19 ^d^	2.41 ± 0.06 ^c^	133.35 ± 1.58 ^b^
8CN	8.00 ± 0.41 ^d^	24.87 ± 0.26 ^e^	2.52 ± 0.07 ^c,d^	139.34 ± 0.70 ^c^

Within column, different superscript letters indicate significant differences among samples (*p* < 0.05; ANOVA, Fisher’s LSD test). Abbreviations: CW—initial conventional wine; CN—nanofiltration retentate of conventional wine; 1—2.5 MPa with cooling; 2—3.5 MPa with cooling; 3—4.5 MPa with cooling; 4—5.5 MPa with cooling; 5—2.5 MPa without cooling; 6—3.5 MPa without cooling; 7—4.5 MPa without cooling; 8—5.5 MPa without cooling.

**Table 6:** Antioxidant activity determined by DPPH, ABTS, FRAP and CUPRAC of initial ecological Cabernet Sauvignon wine and retentates obtained by nanofiltration at 2.5, 3.5, 4.5 and 5.5 MPa with and without cooling.

Sample	DPPH (µmol TE/100 mL)	ABTS (µmol TE/100 mL)	FRAP (µmol TE/100 mL)	CUPRAC (µmol TE/100 mL)
EW	14.77 ± 0.72 ^e^	33.46 ± 0.59 ^e^	3.10 ± 0.13 ^e^	170.85 ± 1.53 ^h^
1EN	5.80 ± 0.39 ^a^	24.16 ± 0.16 ^a^	2.22 ± 0.14 ^b^	107.24 ± 0.45 ^b^
2EN	6.45 ± 0.35 ^a^	24.14 ± 0.23 ^a^	2.34 ± 0.19 ^b,c^	112.96 ± 1.65 ^c^
3EN	8.78 ± 0.34 ^c^	31.35 ± 0.15 ^d^	2.45 ± 0.05 ^c^	138.51 ± 0.38 ^f^
4EN	11.15 ± 0.03 ^d^	31.50 ± 0.17 ^d^	2.62 ± 0.01 ^d^	149.84 ± 1.23 ^g^
5EN	5.77 ± 0.38 ^a^	24.28 ± 0.15 ^a^	1.96 ± 0.02 ^a^	101.44 ± 1.74 ^a^
6EN	5.94 ± 0.49 ^a^	25.58 ± 0.07 ^b^	2.12 ± 0.03 ^b^	127.67 ± 1.06 ^d^
7EN	6.99 ± 0.05 ^b^	27.96 ± 0.11 ^c^	2.42 ± 0.09 ^c^	133.87 ± 1.35 ^e^
8EN	7.11 ± 0.11 ^b^	31.58 ± 0.17 ^d^	2.66 ± 0.06 ^d^	136.37 ± 1.80 ^e,f^

Within column, different superscript letters indicate significant differences among samples (*p* < 0.05; ANOVA, Fisher’s LSD test). Abbreviations: EW—initial ecological wine; EN—nanofiltration retentate of ecological wine; 1—2.5 MPa with cooling; 2—3.5 MPa with cooling; 3—4.5 MPa with cooling; 4—5.5 MPa with cooling; 5—2.5 MPa without cooling; 6—3.5 MPa without cooling; 7—4.5 MPa without cooling; 8—5.5 MPa without cooling.

**Table 7:** Correlation coefficients (*r*) between TPC, TFC, MAC and antioxidant activities obtained by DPPH, ABTS, FRAP and CUPRAC method in conventional and ecological Cabernet Sauvignon wine and their NF retentates at 2.5, 3.5, 4.5 and 5.5 MPa, with cooling and without cooling.

	Conventional Wine			Ecological Wine		
	TPC	TFC	MAC	TPC	TFC	MAC
DPPH	0.934	0.948	0.941	0.943	0.814	0.890
ABTS	0.949	0.887	0.961	0.869	0.869	0.787
FRAP	0.962	0.929	0.983	0.965	0.914	0.960
CUPRAC	0.769	0.759	0.901	0.954	0.924	0.882

**Table 8:** CIELab parameters of initial conventional Cabernet Sauvignon wine and retentates obtained by nanofiltration at 2.5, 3.5, 4.5 and 5.5 MPa, with and without cooling.

Sample	L*	a*	b*	°h	C*	ΔE*
CW	19.70 ± 0.01 ^a^	1.98 ± 0.03 ^b^	1.14 ± 0.03 ^a^	35.80 ± 0.64 ^c^	1.94 ± 0.02 ^a^	-
1CN	19.96 ± 0.04 ^b^	1.95 ± 0.03 ^b^	1.16 ± 0.03 ^a^	29.38 ± 0.28 ^a^	2.25 ± 0.04 ^b^	0.26 ± 0.02 ^a^
2CN	19.96 ± 0.02 ^b^	1.95 ± 0.02 ^b^	1.12 ± 0.03 ^a^	29.70 ± 0.22 ^a^	2.24 ± 0.04 ^b^	0.26 ± 0.02 ^a^
3CN	19.98 ± 0.01 ^b^	2.05 ± 0.04 ^b^	1.15 ± 0.01 ^a^	31.11 ± 0.39 ^b^	2.28 ± 0.02 ^b^	0.29 ± 0.02 ^a,b^
4CN	19.99 ± 0.01 ^b^	2.02 ± 0.04 ^b^	1.14 ± 0.02 ^a^	31.17 ± 0.22 ^b^	2.30 ± 0.02 ^b^	0.30 ± 0.03 ^b^
5CN	20.00 ± 0.03 ^b^	1.86 ± 0.03 ^a^	1.14 ± 0.02 ^a^	29.24 ± 0.32 ^a^	2.31 ± 0.03 ^b^	0.32 ± 0.03 ^b^
6CN	20.00 ± 0.01 ^b^	1.87 ± 0.03 ^a^	1.14 ± 0.03 ^a^	29.60 ± 0.33 ^a^	2.30 ± 0.03 ^b^	0.32 ± 0.01 ^b^
7CN	20.00 ± 0.02 ^b^	1.86 ± 0.02 ^a^	1.14 ± 0.02 ^a^	30.60 ± 0.32 ^b^	2.42 ± 0.01 ^c^	0.32 ± 0.02 ^b^
8CN	20.04 ± 0.04 ^b^	1.83 ± 0.02 ^a^	1.15 ± 0.01 ^a^	30.71 ± 0.39 ^b^	2.42 ± 0.02 ^c^	0.37 ± 0.01 ^c^

Significant differences (*p* < 0.05) between samples are indicated by different superscript letters within the column (ANOVA. Fisher’s LSD test). Abbreviations: CW—initial conventional wine; CN—nanofiltration retentate of conventional wine; 1—2.5 MPa with cooling; 2—3.5 MPa with cooling; 3—4.5 MPa with cooling; 4—5.5 MPa with cooling; 5—2.5 MPa without cooling; 6—3.5 MPa without cooling; 7—4.5 MPa without cooling; 8—5.5 MPa without cooling.

**Table 9:** CIELab parameters of initial ecological Cabernet Sauvignon wine and retentates obtained by nanofiltration at 2.5, 3.5, 4.5 and 5.5 MPa, with and without cooling.

Sample	L*	a*	b*	°h	C*	ΔE*
EW	19.70 ± 0.01 ^a^	2.15 ± 0.02 ^b^	1.07 ± 0.01 ^a^	33.54 ± 0.32 ^b^	1.58 ± 0.03 ^a^	-
1EN	20.28 ± 0.02 ^b^	2.12 ± 0.03 ^b^	1.05 ± 0.04 ^a^	30.51 ± 0.28 ^a^	2.34 ± 0.04 ^b^	0.58 ± 0.01 ^a^
2EN	20.29 ± 0.01 ^b^	2.17 ± 0.01 ^b^	1.03 ± 0.01 ^a^	29.98 ± 0.22 ^a^	2.29 ± 0.02 ^b^	0.59 ± 0.02 ^a^
3EN	20.31 ± 0.01 ^b^	2.14 ± 0.02 ^b^	1.06 ± 0.04 ^a^	30.31 ± 0.39 ^a^	2.56 ± 0.02 ^c^	0.61 ± 0.02 ^a,b^
4EN	20.33 ± 0.02 ^b^	2.13 ± 0.01 ^b^	1.07 ± 0.06 ^a^	29.92 ± 0.22 ^a^	2.56 ± 0.01 ^c^	0.63 ± 0.02 ^b^
5EN	20.29 ± 0.01 ^b^	1.93 ± 0.03 ^a^	1.05 ± 0.02 ^a^	30.06 ± 0.32 ^a^	2.27 ± 0.03 ^b^	0.63 ± 0.03 ^b^
6EN	20.32 ± 0.05 ^b^	1.97 ± 0.02 ^a^	1.04 ± 0.01 ^a^	30.47 ± 0.33 ^a^	2.27 ± 0.03 ^b^	0.65 ± 0.03 ^b^
7EN	20.31 ± 0.01 ^b^	1.94 ± 0.03 ^a^	1.07 ± 0.01 ^a^	30.30 ± 0.32 ^a^	2.51 ± 0.04 ^c^	0.65 ± 0.02 ^b^
8EN	20.32 ± 0.01 ^b^	1.96 ± 0.03 ^a^	1.05 ± 0.01 ^a^	30.45 ± 0.39 ^a^	2.55 ± 0.02 ^c^	0.65 ± 0.02 ^b^

Significant differences (*p* < 0.05) between samples are indicated by different superscript letters within the column (ANOVA. Fisher’s LSD test). Abbreviations: EW—initial ecological wine; EN—nanofiltration retentate of ecological wine; 1—2.5 MPa with cooling; 2—3.5 MPa with cooling; 3—4.5 MPa with cooling; 4—5.5 MPa with cooling; 5—2.5 MPa without cooling; 6—3.5 MPa without cooling; 7—4.5 MPa without cooling; 8—5.5 MPa without cooling.

## Data Availability

Not available.
